# Adolescents' Experiences of Hate Speech and Psychological Needs: A Reflexive Thematic Analysis

**DOI:** 10.1002/jad.70169

**Published:** 2026-04-20

**Authors:** Tomas Jungert, Camilla Forsberg, Robert Thornberg, Lisa Nordlund, Emelie Hansen, Elisa Lagerstedt, Coralie Levesque

**Affiliations:** ^1^ Department of Psychology Lund University Lund Sweden; ^2^ Department of Behavioural Sciences Linköping University Linköping Sweden; ^3^ Département d'organisation et ressources humaines Université du Québec à Montréal Montréal Canada

**Keywords:** adolescents, hate speech, psychological needs, self‐determination theory

## Abstract

**Introduction:**

Adolescents are increasingly exposed to hate speech in both online and offline contexts, yet limited research has examined how such exposure is experienced and how it relates to adolescents' psychological needs and well‐being. Drawing on Self‐Determination Theory (SDT), this study explores how adolescents make sense of hate speech and how these experiences affect autonomy, competence, and relatedness in everyday peer and digital environments.

**Methods:**

Ten focus groups were conducted with 46 Swedish upper secondary school students (girls and boys) aged 16–19 years. Data were analyzed using reflexive thematic analysis within a critical realist framework, allowing for an in‐depth exploration of shared patterns of meaning across school‐based and online contexts.

**Results:**

Four interrelated themes were developed. *Omnipresence of hate speech* captured adolescents' experiences of hate speech as difficult to avoid and emotionally draining. *Ambiguity of hate speech* highlighted challenges in distinguishing hate speech from jokes, memes, or peer‐based humor, complicating moral evaluation and response. *Conformity to hate norms* illustrated how social pressures encouraged tolerance of or participation in hostile talk to maintain belonging, often at the expense of autonomy. Finally, *online‐specific dynamics* showed how anonymity and normalization facilitated hostile behavior, discouraged intervention, and undermined adolescents' sense of competence.

**Conclusions:**

Adolescents experience hate speech as ambiguous, normalized, and difficult to resist, with clear implications for their autonomy, competence, and relatedness. The findings demonstrate how exposure to hate speech can shift motivation toward controlled regulation or disengagement, highlighting the importance of interventions that strengthen adolescents' psychological needs in both peer and digital contexts.

Hate speech has become an increasingly visible form of aggression in adolescents' everyday social environments, particularly online. Studies indicate that around half of young people have experienced online hate speech (Obermaier and Schmuck 2022), and a majority of youth across Western countries (65%–78%) have witnessed it in the past 3 months (Reichelmann et al. 2021). Such exposure often elicits strong emotional responses, including anger, sadness, and shame. A systematic review among youth aged 12–21 revealed that witnessing hate speech was common (26%–69%), while rates of victimization (7%–24%) and perpetration (5%–32%) varied (Kansok‐Dusche et al. 2023). Despite growing concern, research on hate speech among Swedish adolescents remains limited (RBUF n.d.).

Aggression is commonly defined as behavior intended to harm another person (Bushman and Anderson 2001), including physical, verbal, and relational forms. Hate speech can be seen as a specific form of verbal and relational aggression. While it shares features with bullying, such as intent to harm and a social power imbalance, it differs in that bullying typically involves repeated acts toward individuals, whereas hate speech targets social groups and may occur as isolated incidents (Blaya et al. 2022; Wachs et al. 2019). Moreover, whereas bullying often degrades personal traits, hate speech devalues group‐based characteristics such as ethnicity or religion (Bedrosova et al. 2022; Kansok‐Dusche et al. 2023).

Hate speech is generally described as an expression that incites or justifies hostility, violence, or discrimination based on group characteristics (Cohen‐Almagor 2011; Europe 2025; Kansok‐Dusche et al. 2023). In Sweden, the legal concept of *agitation against a population group* (Sveriges riksdag 2025) applies only to certain protected groups, highlighting the need for research beyond legal definitions. At the same time, prior research has emphasized that the boundaries between hate speech, bullying, insults, and humor are often blurred in adolescents' everyday interactions. What constitutes hate speech from a scholarly or legal perspective may not fully align with how young people themselves interpret and label such experiences. In peer contexts, expressions that target group characteristics may be framed as jokes or banter. This makes it difficult to distinguish between general peer aggression and group‐based hostility.

In light of this ambiguity, the present study does not seek to identify hate speech as a strictly defined or objectively bounded phenomenon. Rather, it explores adolescents' subjective understandings and lived experiences of what they themselves perceive and describe as hate speech. This approach captures how boundaries between hate speech, bullying, and everyday interactions are negotiated in practice. However, it also implies that the findings may reflect a broader spectrum of peer aggression, not limited to group‐based hate speech in a strict sense.

Adolescence is a period in which peer acceptance and group belonging are central, making exposure to hate speech particularly consequential. To capture these mechanisms, the present study draws on Self‐Determination Theory (SDT).

## 1 Theoretical Framework

This study draws on SDT (Ryan and Deci 2017, 2024) to explore how adolescents understand and experience hate speech. SDT posits three basic psychological needs, autonomy, competence, and relatedness, essential for growth and well‐being. When thwarted, maladaptive outcomes such as alienation or disengagement may occur (Deci and Ryan 2000; Ryan et al. 2022). Hate speech undermines these needs by targeting adolescents' social identity, belonging, and self‐worth.

Exposure is unevenly distributed and often linked to gender, migration background, religion, or queer identity (Obermaier and Schmuck 2022; Oksanen et al. 2014). Online activity and sharing personal information increase risk, while parental mediation strategies can moderate it (Oksanen et al. 2014; Wachs et al. 2021; Wachs et al. 2021). Offline contexts, including school climate, also influence exposure, highlighting continuity between online and in‐person experiences (Räsänen et al. 2016; Wachs et al. 2023).

From an SDT perspective, hate speech threatens autonomy, relatedness, and competence, lowering well‐being (Keipi et al. 2018; Wright and Wachs 2019). Social Cognitive Theory (Bandura 2001) and moral disengagement (Bandura et al. 1996) explain how adolescents justify or tolerate harm, e.g., “it's just a joke,” enabling participation without guilt. Empirical studies linked moral disengagement to aggression and hate speech (Castellanos et al. 2024; Oksanen et al. 2014; Wachs et al. 2024; Wachs et al. 2022).

Moral disengagement refers to the cognitive and social mechanisms that allow individuals to justify, minimize, or disregard the moral implications of harmful actions. These mechanisms function across four domains: reinterpreting one's behavior, displacing or diffusing responsibility, distorting or downplaying consequences, and dehumanizing or blaming the victim. In the context of hate speech, individuals may, for example, claim that “it was just a joke,” minimize harm (“no one was really hurt”), or shift responsibility (“everyone talks that way”). Such rationalizations enable participation in or tolerance of hostile communication without experiencing moral conflict or guilt.

Empirical research has consistently demonstrated associations between moral disengagement and various forms of aggression, bullying, and bystander behavior (Killer et al. 2019; Lo Cricchio et al. 2021; Thornberg and Jungert 2014). More recently, several quantitative studies have linked moral disengagement to hate speech perpetration, often as a mediating or explanatory mechanism.

Although qualitative and mixed‐method studies have examined adolescents' lived experiences of hate speech (Ballaschk et al. 2021; Krause et al. 2021; Vaught 2012), this research has primarily focused on describing exposure, emotional responses, and social contexts. Less attention has been given to the motivational processes through which such experiences affect adolescents' well‐being and social functioning. The present study addresses this gap by applying SDT in a qualitative design to examine how adolescents interpret and respond to hate speech in relation to autonomy, competence, and relatedness. By combining SDT with moral disengagement, the study provides a theoretically informed account of how adolescents navigate the tension between social belonging and authentic self‐expression in contexts where hate speech is normalized.

## 2 Aim and Research Questions

Building on the theoretical framework outlined above, the present study seeks to deepen the understanding of adolescents' lived experiences of hate speech through the lens of SDT. Specifically, it aims to examine how Swedish upper secondary school students conceptualize hate speech, its underlying mechanisms, and how such experiences relate to the satisfaction and frustration of basic psychological needs and overall well‐being.

The study addresses the following research questions:

1. How do adolescents describe and conceptualize hate speech?
2. How do adolescents perceive that exposure to hate speech affects the satisfaction of their basic psychological needs?

## 3 Method

### 3.1 Design and Approach

Ten focus groups were conducted to explore adolescents' experiences of hate speech. Focus groups were chosen to facilitate interaction and collective sense‐making, allowing participants to reflect on and negotiate shared norms, meanings, and experiences related to hate speech in peer contexts. This approach was considered particularly suitable for capturing socially embedded and relational aspects of the phenomenon, thereby generating rich and nuanced data. Discussions were analyzed using reflexive thematic analysis (Braun and Clarke 2021), a method suitable for identifying patterns of meaning. The study adopts a critical realist epistemology, recognizing that data requires interpretation to understand the underlying structures of the phenomenon. Hate speech was treated as a socially constructed and context‐dependent phenomenon, experienced as real in adolescents' everyday lives, but requiring interpretation to understand its boundaries, mechanisms, and impact.

In line with the exploratory aim of the study, hate speech was not operationalized as a strictly predefined construct. Instead, the study focused on participants' subjective understandings and lived experiences of what they themselves perceived and described as hate speech. During data collection, no formal definition was imposed; rather, participants were encouraged to define and discuss the phenomenon in their own terms. This approach enabled the analysis to capture how adolescents interpret and negotiate the boundaries between hate speech, bullying, insults, and humor in everyday peer contexts. As such, the findings may reflect a broader spectrum of peer aggression, including but not limited to group‐based hate speech in a strict sense.

### 3.2 Participants

The dataset was obtained from six upper secondary schools located in diverse boroughs and cities in Sweden, including both large urban areas and smaller municipalities. Schools were selected to reflect variation in socioeconomic composition, academic achievement (Skolverket 2024), and size (approximately 400 to 1000 students), and from small to larger municipalities. Institutional consent was obtained from principals and schoolteachers, and students were invited by the research team to participate through both written and verbally informed assent in focus groups.

In all, 46 students (26 female, 20 male) aged 16–19 participated. Ten focus groups were held across the six schools. Most participants were enrolled in social science programs, with others in technical, aesthetic, or sports tracks. Prior exposure to hate speech was not required for participation.

The number of focus groups was guided by the principle of thematic saturation, with data collection continuing until no substantially new patterns of meaning were identified.

While the participating schools reflected variation in socioeconomic and demographic composition, detailed individual‐level data on migration background, socioeconomic status, and digital media use were not systematically collected.

Although digital media use was discussed in the focus groups, participants' usage patterns were not collected in a standardized way. These characteristics could therefore not be examined analytically in the present study.

### 3.3 Data Generation

Focus groups included three to seven participants (*M* = 5.75) and followed a semi‐structured guide covering their home, school, and leisure contexts, how they define hate speech, their experiences of exposure, and the contexts in which they encountered hate speech (e.g., in school spaces, peer interactions, and across social media platforms). Example questions included: “What does hate speech mean to you, and how would you describe what it looks like in practice?” and “Can you describe a situation where you have seen or heard hate speech, and how such experiences affect people your age?” Sessions lasted 40–75 min (*M* = 55). Sessions were conducted in private rooms at the schools.

Interviews were conducted in Swedish, audio‐recorded, and transcribed verbatim. Transcripts were anonymized and reviewed for accuracy. The focus groups were conducted by two final‐year psychology students and one senior researcher, all members of the research team.

### 3.4 Data Analysis

Data were analyzed following Braun and Clarke's six‐phase reflexive thematic analysis (Braun and Clarke 2021). Coding was abductive, involving an iterative movement between empirical data and theoretical concepts. Initial codes were generated inductively from participants' responses, while subsequent coding incorporated relevant theoretical constructs from SDT (Ryan and Deci 2017, 2024) and moral disengagement (Bandura et al. 1996). This allowed us to capture patterns emerging from the data while also considering how existing theory could help interpret underlying mechanisms of hate speech and its social normalization. Thematic analysis was primarily conducted by the interviewers, with input and refinement from the first author, to ensure coherence and analytic rigor. Themes were iteratively developed, reviewed, refined, and defined, resulting in four main themes. Findings were synthesized into a coherent narrative, with participant quotes edited for clarity and irrelevant content removed while maintaining authenticity.

The analysis was primarily inductive in its initial phases, focusing on participants' own descriptions and meanings. Concepts from SDT were not used as predefined coding categories but were introduced in later stages of the analysis as sensitizing concepts to support the interpretation of emerging patterns. This means that the themes were developed from the data, and subsequently interpreted in relation to autonomy, competence, and relatedness, rather than being derived deductively from SDT.

Coding was conducted by multiple members of the research team. The focus group interviewers (two final‐year psychology students and the first author) initially engaged in coding the data, generating preliminary codes based on their close familiarity with the material. One additional senior researcher subsequently conducted an independent coding of the dataset, providing a more distanced and theoretically informed perspective. A further senior researcher reviewed the developing analysis, including codes, candidate themes, and additional excerpts from the data to challenge and refine interpretations. Themes were developed through an iterative and reflexive process involving ongoing discussions within the research team. Rather than aiming for consensus in a positivist sense, these discussions focused on exploring different interpretations, questioning assumptions, and strengthening the coherence and depth of the analysis, in line with principles of reflexive thematic analysis (Braun and Clarke 2021).

Reflexivity was considered throughout the research process. The research team consisted of both junior and senior researchers with backgrounds in psychology. The interviewers' close involvement in data collection likely shaped initial interpretations through their proximity to participants' accounts, while the involvement of senior researchers in later stages contributed to more distanced and theoretically informed perspectives. All researchers had prior familiarity with research on hate speech, which may have increased sensitivity to certain patterns in the data. To address this, the team engaged in ongoing discussions to reflect on assumptions, challenge interpretations, and consider alternative readings, aiming to remain open to the complexity and ambiguity in participants' accounts.

### 3.5 Ethical Considerations

The study followed established ethical guidelines for research involving human participants. Institutional consent was obtained from school principals, and students provided informed assent. Participation was voluntary, data were anonymized, and all recordings and transcripts were securely stored. Ethical approval was obtained from the Swedish Ethical Review Authority (Dnr 2023‐07602‐01, decision date 2024‐02‐12).

## 4 Results

Through reflexive thematic analysis, four themes were developed. The first two, *Blurred boundaries of hate speech* and *Omnipresence of hate speech* address the first research question regarding how adolescents describe hate speech. The remaining two, *Conformity to hate speech* and *Normalization of online hate speech,* address the second research question concerning how hate speech affects adolescents' experiences and well‐being.

### 4.1 How Do Adolescents Describe Hate Speech?

#### 4.1.1 Blurred Boundaries of Hate Speech

Participants consistently described hate speech as a difficult concept to define. They often blurred the lines between hate speech and other forms of aggression, such as gossip, insults, or bullying. Subtle expressions, such as GIFs, memes, usernames, facial expressions, or comparisons, were also recognized as potentially hateful, further complicating definitions. One participant explained, “Sometimes it's not directly a hate comment, but they send GIFs or similar things that are meant as hate comments.” Similarly, humor and peer context mattered. Comments that were considered acceptable among friends might be perceived as hateful in other contexts.

Several participants emphasized that what might appear as hate speech to outsiders was often seen within the group as ordinary jokes or playful chatting. They described how words that originally carried hostile meanings had, in their peer interactions, become part of everyday humor rather than expressions intended to harm. As one participant put it, “Many people don't see hate comments as hate comments, but just as jokes… they joke around and don't realize that maybe it's not such an appropriate joke.” Another participant admitted, “Sometimes it can actually be a bit funny, I have to admit.”

These accounts suggest that illustrate how normalization of hate speech can occur at a linguistic level, where meanings shift through repeated, humorous use. Within the in‐group, such expressions are often detached from their original derogatory intent, while for outsiders they might still appear offensive. This linguistic desensitization contributes to the blurred boundaries between hate speech, humor, and ordinary communication, making it even harder for adolescents to determine when speech becomes hateful.

#### 4.1.2 Omnipresence of Hate Speech

The second theme emphasized the ubiquity of hate speech. Participants described it as pervasive, both online and offline, targeting anyone regardless of behavior, appearance, or status. Hate speech could be directed at minor or major traits, such as family, lifestyle, talents, possessions, political opinions, or physical appearance. Participants stressed that even those who conform to social ideals are not immune. As one participant noted: “Even people who meet the ideals can receive hate…/…/for being too successful, and someone else gets hate for not looking like them.”

Participants also highlighted that children are vulnerable. Comments directed at minors, such as in parental social media posts, can attract abusive responses, illustrating that no one is fully safe from hate speech: “Even children… someone posts a picture of a child dancing on Instagram, and the comments are all just hate: ‘Kill yourself,’ ‘Where are your parents?’ It's crazy.” The theme captures the sense that, particularly online, hate speech is difficult to avoid and can emerge across diverse contexts and targets.

#### 4.1.3 Conformity to Hate Speech

Participants described how they sometimes adjusted their behavior to fit the group's way of speaking, even when this involved comments or jokes about different social groups or individuals unrelated to themselves. Laughing along, pretending something was funny, or treating hostile remarks as “normal” were strategies used to avoid standing out and to be accepted. One participant explained, “Even if nobody in the group is directly affected, if someone makes a comment about a minority, you laugh along because that's what everyone does.”

This adaptation reflects how adolescents can feel pressured to conform to social norms, even when these include subtle forms of hate speech. Participants emphasized that this conformity often limited their ability to act authentically or express their true opinions, highlighting difficulties in expressing their own views openly. Many participants said that they did not necessarily agree with the jokes or comments but felt they had to participate to maintain their place in the group.

At the same time, participants noted that conforming to group norms reinforced a sense of belonging. Laughing along or participating in group humor, even when it involved hostile or exclusionary content, created a stronger sense of belonging. The desire to fit in and avoid social exclusion motivated much of this behavior. One participant summarized: “You do it because you want to belong. Even if you don't really think it's funny, you laugh because everyone else does, and you don't want to be left out.”

Some participants described a nuanced tension: they could maintain social connections and feel accepted while simultaneously suppressing their personal views, illustrating the tension between expressing personal views and maintaining social belonging. Participants sometimes described adjusting their behavior in ways that may help to maintain a sense of social competence, particularly in navigating group dynamics. By following the group's lead, even passively, they could feel capable of navigating the social dynamics and avoid standing out as “wrong” or socially inadequate. As one participant explained:

Because it's also like, the people who say those things probably wouldn't say them if they were on their own. But it's also kind of about peer pressure, like, your friends think a certain way, and you want to feel like you fit in, that kind of thing.

Another participant captured the competence‐related aspect more directly, adding, “Yeah, to feel like you're best at it”.

Feelings related to managing social interactions appeared to be less directly affected, though some participants mentioned feeling unsure or unskilled in navigating these social dynamics, especially in groups where hostile humor or exclusionary comments were common.

Overall, the theme highlights how peer pressures and group norms can subtly shape behavior. Even in the absence of direct participation in hate speech, adolescents may internalize the group's language and humor, adjusting their behavior in ways that compromise authenticity but strengthen social bonds. This suggests that maintaining social acceptance can support a sense of social effectiveness, even when it comes at the cost of expressing one's own views fully.

#### 4.1.4 Normalization of Online Hate Speech

Participants described the online environment as one where hate speech is pervasive, easily produced, and rarely met with consequences. Anonymity emerged as a central mechanism lowering the threshold for hostile expression. Several participants emphasized that anonymity enables individuals to act as “invisible perpetrators,” expressing hate without having to take responsibility or risk social sanctions. At the same time, anonymity also facilitates becoming an “invisible bystander,” allowing users to witness hate without being seen or expected to intervene. This dual invisibility was described as reinforcing both hostility and passivity, contributing to a broader culture in which hate speech is tolerated.

Another dimension participants highlighted was the “online flooding of hate speech.” The sheer volume of hostile comments circulating on platforms, particularly in comment sections and algorithmically amplified content feeds, was perceived as normalizing hostility by making it appear constant and unavoidable. As one participant noted, “You see someone getting hate on TikTok, and it's horrible, but you don't react because it's just constant.” This continuous exposure created a sense of desensitization, where hate speech became expected rather than exceptional, further reducing the motivation to intervene.

These dynamics appeared to reduce adolescents' motivation to stand up against hate speech. Participants described feeling external pressures to adapt to dominant online norms, either by joining in hostile commentary to fit in or by remaining silent to avoid backlash. These pressures made their actions feel driven by social expectations rather than personal values, as actions were driven less by personal values and more by social expectations or fear of judgment. Many also reported uncertainties about how to respond effectively, suggesting uncertainty about how to respond effectively. This combination could contribute to withdrawal, either through passive scrolling or avoidance of online interactions altogether. One participant explained, “Sometimes I just scroll past everything because it's too much, and I don't want to get involved.”

Overall, the accounts suggest that anonymity, invisibility, and the flooding and normalization of hate speech create an environment that may undermine adolescents' sense of agency and social competence. These conditions may contribute to adolescents' withdrawal from responding to hate speech online and highlight how digital contexts can shape motivation in ways that risk reinforcing harmful social norms.

## 5 Discussion

This study explored how Swedish upper secondary school students conceptualize hate speech and how their experiences of such hostility relate to psychological need satisfaction and well‐being. In our reflexive thematic analysis, we identified four themes. The blurred boundaries of hate speech and Omnipresence of hate speech captured how adolescents define and perceive hate speech. Conformity to hate speech and normalization of online hate speech illustrated the mechanisms through which exposure affects basic psychological needs and social functioning. Together, these themes reveal that hate speech is perceived not as an isolated act but as a diffuse, normalized feature of adolescents' everyday environments, both online and offline, and that it has implications for adolescents' autonomy, competence, and relatedness.

### 5.1 How Adolescents Conceptualize Hate Speech

#### 5.1.1 Blurred Boundaries of Hate Speech

Participants described hate speech as a fluid, context‐dependent phenomenon, with blurred boundaries between hate speech, bullying, and general aggression (Kansok‐Dusche et al. 2023; Titley 2014). They emphasized that hate speech can be expressed indirectly, such as through jokes, memes, or facial expressions, and that both intent and interpretation determine whether an act is perceived as hateful. Such accounts align with studies suggesting that covert hostility and microaggressions reinforce power hierarchies while remaining socially accepted (Sue et al. 2007). This ambiguity also points to the moral and emotional role of humor in social interactions. As Morgan and DiFranco (2021) argue, humor can suspend ordinary moral evaluations and allow individuals to laugh at or with others without perceiving their actions as wrong. In this sense, adolescents' descriptions of “just joking” reveal how amusement can blur moral boundaries and soften the perceived harm of hostile language. Within peer groups, repeated humorous use of offensive words may dilute them of their original derogatory meaning, transforming hate speech into normalized, playful talk. From this perspective, normalization of hate speech involves not only moral and emotional processes but also linguistic and cultural ones that redefine what is considered acceptable speech.

From a social cognitive perspective (Bandura 2001), adolescents' descriptions of hate speech as “just joking” can be interpreted as an instance of moral disengagement, specifically in terms of reinterpreting this negative behavior through euphemistic labeling. By framing hostile or derogatory expressions as “jokes”, “banter,” or “playful talk,” they linguistically sanitize their or their peers' behavior and downplay its harmfulness. This makes it easier to engage in and tolerate hate speech without experiencing moral self‐sanction such as guilt, shame, or remorse. This normalization process may also have implications for adolescents' psychological experiences. If repeated exposure leads hostile expressions to be interpreted as “normal” or “just joking,” normalization may moderate the relationship between exposure to hate speech and psychological need frustration. On one hand, perceiving hostile remarks as playful might buffer adolescents from experiencing threat or exclusion; on the other, normalization could mask harm and make it more difficult for youth to recognize and respond to subtle forms of need frustration. These possibilities suggest that future work should examine when and how normalization attenuates or amplifies the impact of hate speech on adolescents' basic psychological needs. Adolescents' reflections, therefore, show that understanding hate speech requires attention to the subtle moral judgments, linguistic practices, and emotional interpretations that give meaning to everyday interactions.

#### 5.1.2 Omnipresence of Hate Speech

The theme Omnipresence of hate speech conveyed adolescents' sense of hate speech as universal and inescapable. Participants described how hostility could be directed at anyone, for various reasons such as appearance, success, opinions, or simply differences. This supports prior findings that online hate speech is pervasive and targets diverse groups (Obermaier and Schmuck 2022; Reichelmann et al. 2021). Hate speech was seen as part of the social “background noise” of adolescence, both online and offline. From a social‐cognitive perspective (Bandura 2001), this normalization can be understood as a collective form of *moral disengagement*, where repeated exposure leads to desensitization and the minimization of moral responsibility. As harmful communication becomes routine, self‐sanctions are weakened, and adolescents may learn to rationalize or overlook hate speech to maintain group belonging or social status. The normalization of hate speech thus operates both as a psychological adaptation and a learned social practice.

### 5.2 How Hate Speech Affects Need Satisfaction and Motivation

To further interpret how these experiences relate to adolescents' motivation and well‐being, the following section draws on SDT. It is important to note that participants did not explicitly refer to psychological needs such as autonomy, competence, or relatedness. Rather, these constructs are used here as interpretative tools to make sense of patterns in participants' accounts. The analysis, therefore, reflects a theoretically informed reading of the data, rather than a direct mapping of participants' language onto SDT constructs.

#### 5.2.1 Conformity to Hate Speech

Adolescents described feeling pressure to adapt to peer norms that tolerate or trivialize hate speech. Joining in laughter, remaining silent, or avoiding confrontation were strategies used to preserve inclusion and social harmony. This dynamic can be understood as reflecting a tension between relatedness and autonomy, as described in SDT (Ryan and Deci 2017, 2024). Conforming to group norms may temporarily support a sense of belonging while simultaneously constraining authentic self‐expression, a core aspect of autonomy in SDT. Recent research on bystanders in school bullying similarly shows that adolescents often experience a loss of authenticity when remaining passive, describing a disconnect between their internal moral stance and their outward behavior (Olsson et al. 2025). These patterns mirror moral disengagement processes (Bandura et al. 1996), especially diffusion of responsibility and euphemistic labeling. Similar findings have been reported in studies linking moral disengagement to bullying (Bjärehed et al. 2021; Thornberg and Jungert 2014; Thornberg et al. 2023) and online aggression (Chen and Chen 2025; Falla et al. 2021; Runions and Bak 2015). Adolescents' accounts illustrate how moral reasoning and motivational processes intertwine. They also show how the pursuit of social acceptance can override internalized moral standards and undermine autonomous motivation to stand up against hate speech.

This dynamic may also be interpreted through a social identity perspective (Tajfel and Turner 2004), where aligning with peer norms can reinforce a sense of ingroup belonging. In this sense, conforming to or tolerating hate speech may function as a way to maintain group membership and social standing, even when it conflicts with personal values. Importantly, this tension between autonomy and relatedness raises broader developmental questions. Although peer conformity pressures are very high in adolescence (Laursen and Veenstra 2021; Santor et al. 2000), similar autonomy–relatedness trade‐offs may also appear in adulthood. Adults also adjust their expressed opinions, humor, or silence in workplaces and social groups to maintain belonging. This suggests that these processes may not be unique to youth (Duarte and Thompson 1999; Ju et al. 2019; Plester et al. 2022). However, the salience of peer approval during adolescence likely heightens the intensity of the autonomy–relatedness conflict, making authenticity especially vulnerable in this developmental period.

Age‐related differences in social priorities may shift which needs are most impacted. For example, adults may experience stronger competence‐related frustration when navigating hostile or exclusionary group norms, particularly in professional contexts where social performance, expertise, or career implications are salient. This mirrors the mechanism in adolescence described above, where alignment with the ingroup can temporarily protect a sense of competence (Gini 2006). Future research comparing adolescents and adults could help clarify how the developmental stage shapes which psychological needs are most sensitive to these social pressures.

#### 5.2.2 Normalization of Online Hate Speech

The digital context appears to intensify these dynamics. Participants highlighted how anonymity and physical distance online increase processes that can be linked to moral disengagement (Bandura 2001), which made it possible for individuals to express hostility without accountability. The absence of direct social feedback weakens empathy and increases the perceived social acceptability of hate speech (Wachs et al. 2022). Our qualitative findings are consistent with the *online disinhibition effect*, whereby anonymity, invisibility, asynchronicity, and reduced accountability lower inhibitions and weaken immediate social sanctions, thereby facilitating hostile expression such as hate speech (Suler 2005).

At the same time, there appears to be what might be termed an *online flooding effect*: frequent exposure to hate speech may reshape perceived norms so that hostility comes to be seen as typical or expected online, which further increases the likelihood that individuals will voice prejudice and join in hate speech (Álvarez‐Benjumea 2023). From a moral disengagement perspective (Bandura 2001), this normalization of online hate speech can be understood in terms of diffusion and displacement of responsibility within large, anonymous online crowds, making each individual feel only minimally accountable for harm.

From an SDT perspective, such environments frustrate both autonomy and competence: adolescents feel pressured to conform to online hostility or withdraw entirely, resulting in controlled regulation or amotivation (Ryan and Deci 2024). Repeated exposure also undermines perceived competence in managing social interactions, contributing to a learned helplessness and moral disengagement pattern. Over time, these experiences may generalize beyond digital contexts, affecting motivation and well‐being more broadly.

### 5.3 Integrating the Findings

Before integrating the findings, it is important to clarify how the conceptual ambiguity surrounding hate speech shapes the interpretation of the results. Because the study focused on adolescents' subjective understandings, participants' accounts often blurred the boundaries between hate speech, bullying, and general peer aggression. This reflects how such phenomena are experienced and negotiated in everyday peer contexts but also means that the findings should not be interpreted as referring exclusively to hate speech in a strict, group‐based sense. Rather, they capture a broader spectrum of hostile and ambiguous social interactions, in which elements of both group‐based hate speech and other forms of peer aggression may be intertwined.

Across the four themes, hate speech emerges as a socially embedded and morally complex phenomenon. Adolescents' accounts suggest that hate speech is sustained through learned patterns of justification and conformity, consistent with Bandura (2001) social cognitive framework of moral disengagement. However, when interpreted through the lens of SDT, these same processes can be seen as reflections of frustrated basic psychological needs. When belonging depends on participating in or tolerating hate speech, the need for relatedness is met at the expense of autonomy and competence, resulting in diminished well‐being and moral agency.

### 5.4 Implications

Hate speech often blends with humor and ordinary peer interactions. This suggests that prevention efforts should address everyday linguistic practices and highlight how jokes or memes can reproduce exclusion. Teaching adolescents about moral disengagement and how to resist its mechanisms may strengthen prevention. Interventions should go beyond individual behavior, targeting motivational and contextual conditions that sustain hostility. The ubiquity of hate speech calls for whole‐school and multi‐level approaches, including positive school climates, classroom norms, adult modeling, and collaboration with parents and communities. Need‐supportive environments, fostering autonomy, empathy, and critical dialogue about group norms, may buffer against moral disengagement and encourage prosocial motivation. Educational programs promoting moral reflection and digital literacy can help adolescents recognize and resist normalization processes that undermine empathy and authenticity.

At a broader level, an ecological perspective emphasizes that reducing hate speech requires coordinated efforts across families, schools, and digital platforms to support autonomy, competence, and relatedness. Strengthening these needs across contexts may help adolescents develop the moral courage and motivation to challenge hate speech rather than accommodate it. These findings may also be relevant for existing school‐based prevention programs targeting hate speech and peer aggression. For example, interventions such as HateLess (Wachs et al. 2023), which promote empathy, classroom climate, and active bystander behaviors, align with the present findings by addressing both social norms and students' psychological needs. Integrating a need‐supportive perspective into such programs may further strengthen their effectiveness.

### 5.5 Limitations and Future Research

The qualitative, context‐specific design limits transferability, as participants were Swedish upper secondary students. Future research could use longitudinal or mixed methods to explore how exposure to hate speech interacts with moral disengagement and need frustration over time. Examining protective factors such as empathy, moral courage, digital citizenship, and need‐supportive teacher practices could illuminate pathways toward resilience. Comparative studies across cultural contexts may clarify how societal and digital systems shape adolescents' experiences and motivational responses to hate speech.

## 6 Conclusion

Adolescents experience hate speech as a pervasive, socially embedded phenomenon that balances belonging with authenticity. Conformity to hostile peer norms supports social inclusion but can frustrate autonomy and competence, reducing well‐being. Moral disengagement and normalization allow adolescents to navigate these interactions, yet social connection often comes at the cost of self‐efficacy and authenticity. Framed by SDT and social‐cognitive perspectives, these findings suggest that addressing hate speech requires need‐supportive social environments rather than only individual moral education, fostering autonomy, genuine relationships, and critical reflection on everyday communication norms.

## Author Contributions

**Tomas Jungert:** conceptualization, funding acquisition; writing – original draft, methodology, writing – review and editing, project administration, supervision, formal analysis. **Camilla Forsberg:** conceptualization; funding acquisition, writing – review and editing, methodology, formal analysis, project administration, data curation. investigation. **Robert Thornberg:** conceptualization; funding acquisition, methodology, writing – review and editing, supervision, project administration, formal analysis. **Lisa Nordlund:** methodology, writing – review and editing, data curation. **Emelie Hansen:** conceptualization, investigation, data curation, formal analysis. **Elisa Lagerstedt:** formal analysis, conceptualization, investigation, data curation. **Coralie Levesque:** writing – review and editing.

## Data Availability

The data that support the findings of this study are available on request from the corresponding author. The data are not publicly available due to privacy or ethical restrictions.
